# Two-Step Thermal Transformation of Multilayer Graphene Using Polymeric Carbon Source Assisted by Physical Vapor Deposited Copper

**DOI:** 10.3390/ma16165603

**Published:** 2023-08-13

**Authors:** Yong Huang, Jiamiao Ni, Xiaoyu Shi, Yu Wang, Songsong Yao, Yue Liu, Tongxiang Fan

**Affiliations:** State Key Laboratory of Metal Matrix Composites, School of Materials Science and Engineering, Shanghai Jiao Tong University, Shanghai 200240, China; yonghuang@sjtu.edu.cn (Y.H.);

**Keywords:** polymeric carbon source, dielectric substrate, two-step thermal transformation, metallic copper catalyst, transfer-free graphene film

## Abstract

Direct in situ growth of graphene on dielectric substrates is a reliable method for overcoming the challenges of complex physical transfer operations, graphene performance degradation, and compatibility with graphene-based semiconductor devices. A transfer-free graphene synthesis based on a controllable and low-cost polymeric carbon source is a promising approach for achieving this process. In this paper, we report a two-step thermal transformation method for the copper-assisted synthesis of transfer-free multilayer graphene. Firstly, we obtained high-quality polymethyl methacrylate (PMMA) film on a 300 nm SiO_2_/Si substrate using a well-established spin-coating process. The complete thermal decomposition loss of PMMA film was effectively avoided by introducing a copper clad layer. After the first thermal transformation process, flat, clean, and high-quality amorphous carbon films were obtained. Next, the in situ obtained amorphous carbon layer underwent a second copper sputtering and thermal transformation process, which resulted in the formation of a final, large-sized, and highly uniform transfer-free multilayer graphene film on the surface of the dielectric substrate. Multi-scale characterization results show that the specimens underwent different microstructural evolution processes based on different mechanisms during the two thermal transformations. The two-step thermal transformation method is compatible with the current semiconductor process and introduces a low-cost and structurally controllable polymeric carbon source into the production of transfer-free graphene. The catalytic protection of the copper layer provides a new direction for accelerating the application of graphene in the field of direct integration of semiconductor devices.

## 1. Introduction

Graphene’s atomic-level thickness and excellent charge carrier properties have made it a highly sought-after material in the field of semiconductor device integration [1,2]. In the past two decades, research has expanded the application scenarios of optoelectronic devices based on dielectric substrates and graphene materials to include virus detection [3,4], gas sensing [5], integrated circuits [6,7], optoelectronic detection [8,9], and other fields. However, the conventional chemical vapor deposition technique based on transition metal foil, used in the synthesis of two-dimensional graphene, poses challenges in achieving compatibility with current semiconductor processes due to the complicated and time-consuming post-physical transfer process [10,11]. Additionally, the transfer process can degrade the intrinsic physical properties of graphene [12]. To overcome these challenges, researchers are actively seeking a transfer-free in situ preparation method for graphene on dielectric substrates [13,14,15] that is effectively compatible with semiconductor device integrated fabrication processes and that has reached different electronic device applications, including Schottky photodetector array [16], quantum Hall device [17], and electrochromic device [18].

Compared with gaseous carbon sources [19], which have high overall costs and storage and transportation risks, solid carbon sources are commonly preferred for their inexpensive availability and controllable structural design [20,21]. Solid carbon sources, such as arc-deposited amorphous carbon [22,23,24], diamond [14], graphite powder [25,26], silicon carbide [27], and small-molecule organic matter [28,29], combined with specific structural designs and metal catalysts have been used to obtain transfer-free graphene with different numbers of layers and for different application scenarios. Self-assembled monolayers (SAMs) with different molecular structures and chemical compositions can be used to prepare transfer-free graphene with different layer numbers and structures on a variety of dielectric substrates [30,31], but the physical performance of the obtained graphene is somewhat disturbed due to the presence of intrinsic heteroatoms such as silicon. In contrast, polymers represented by polymethyl methacrylate (PMMA) provide a more suitable carbon source option for the preparation of transfer-free graphene due to the advantages of spin-coating controllability and the simplicity of chemical composition. In recent years, graphene with different layers has been synthesized on dielectric substrates using polymers of various molecular structures based on metal catalysis [32,33,34,35].

Existing studies on the preparation of transfer-free graphene from polymeric carbon sources mainly focus on nickel as a catalyst, which is a kind of magnetic metal and difficult to use in semiconductor processes. Copper, however, has lower carbon solubilization ability [36] and is in widespread use in semiconductor processes as interconnects or electrodes, making it an attractive alternative. Further research is needed to fully understand the transformation mechanism and chemical evolution of polymeric carbon sources, and the use of copper as a catalyst will advance the field and accelerate the development of graphene-based semiconductor devices. A more comprehensive understanding of the transformation process and the use of more compatible catalysts can result in better performance and increased potential applications for transfer-free graphene film.

In this study, PMMA was used as a representative carbon source to investigate its chemical composition evolution during the two-step thermal transformation process for the growth of transfer-free graphene films. The protective and catalytic effects of a metallic copper layer were examined, and the microscopic transformation process from PMMA to amorphous carbon to graphene was elucidated in detail. The role and surface structure evolution of metallic copper films in the thermal transformation of PMMA were also systematically studied. This research provides important insights for ensuring the successful transformation of PMMA into transfer-free graphene films and offers suggestions for further improving the quality of such films based on polymeric carbon sources. The findings of this work can help to enhance the compatibility of graphene film preparation technology with semiconductor processes and promote the application of graphene films in the integrated fabrication of semiconductor devices.

## 2. Materials and Methods

### 2.1. Specimen Preparation and Thermal Transformation Process

**Figure 1:**
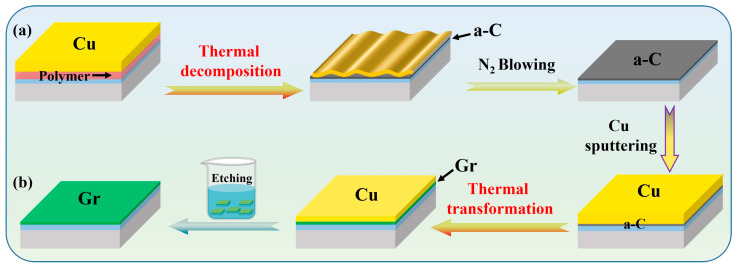
Schematic of transfer-free graphene synthesis by two-step thermal transformation method. (**a**) Flow chart of thermal decomposition of PMMA and copper-assisted amorphous film production on dielectric substrate after nitrogen blowing. The copper film is sputtered on the substrate by magnetron sputtering system and has a thickness of 200 nm. (**b**) Copper-catalyzed graphene film transformation process on dielectric substrate and metal removal after etching. Notes: “a-C” refers to amorphous carbon, while “Gr” refers to graphene.

The dielectric substrate used in this experiment was a 4-inch, 300 nm thermal silicon oxide wafer prepared by the Advanced Electronic Materials and Devices School Platform of Shanghai Jiao Tong University (AEMD-SJTU, Shanghai, China). The specimens were cut into small slices of approximately 1 cm × 1 cm by a diamond cutting knife. To ensure high-quality spin-coating of the polymer solution, hydrophilic treatment was required due to the hydrophobic nature of the wafers in their natural state. The silicon oxide slices were ultrasonically washed in acetone, deionized water, and ethanol for 15 min each, and the liquid residues were blown off using dry nitrogen before each washing procedure. UV treatment was used to improve the wettability of the polymer solution on the silicon oxide slices and the optimized exposure time is ~30 min. PMMA solution with a mass fraction of 0.5 % was then uniformly spin-coated on the treated silicon oxide slices using a homogenizer, with spinning velocity and time of ~6000 rpm and ~60 s, respectively. The Cu films with a thickness of 200 nm were sputtered using a magnetron sputtering system. The thermal transformation experiments were carried out in a tubular furnace with hydrogen gas protection (~100 sccm). The steady-state temperature and holding time were controlled in the range of 400–950 °C and 10–60 min, respectively, and the specific parameters can be seen in the corresponding figure. After the first thermal transformation, the copper film on the specimen’s surface was removed by nitrogen blowing (Figure 1a). For the second thermal transformation, the copper film on the specimen’s surface was chemically etched by marble solution (Figure 1b). The specimens were rinsed several times with deionized water and dried by nitrogen before annealing in a vacuum oven (chamber pressure ~9 × 10^−4^ Torr) at 100 °C for 5 min to remove water molecule adsorption.

### 2.2. Structural and Property Characterization

Thermogravimetric analysis of PMMA solid particles was carried out using the TGA 8000 thermal analyzer produced by PerkinElmer (Waltham, MA, USA). The temperature range for analysis was from 25 °C to 700 °C with a heating rate of 10 °C/min, and the gas flow rate was ~40 mL/min in both air and N_2_. The surface roughness of the specimen was measured using the MFD-300 atomic force microscope manufactured by Oxford Instruments, with a longitudinal resolution of 0.02 nm and a test range of 20 × 20 μm^2^. The microtopography and EDS spectrum of the sample were analyzed using the RISE-MAGNA Raman image-scanning electron microscope (TESCAN, Brno, Czech Republic), with an accelerating voltage of 20 kV, beam current of 200 pA, and optimal focal length of 5 mm. The Raman spectrum and optical photographs of the sample were obtained using the Renishaw inVia Qontor with a laser power of 60 mW, spot size of 0.5 μm, and an acquisition wavenumber range of 1200–2800 cm^−1^, with the surface mapping sweep step size set to 0.5 μm. Chemical bonding information of the heat-transformed products was analyzed by the Mini Flex 600 XPS spectrometer produced by Rigaku (Tokyo, Japan), using monochromaticized Al Kα as the X-ray source, with a test power of 150 W, scanning step size of 1000.0 meV, and residence time of 100 ms. The full spectrum scanning range was 0–1200 eV, while the C 1 s and the Cu 2p_3/2_ spectrum scanning ranges were 273–296 eV and 924–968 eV, respectively. For electrical performance characterization, a four-probe resistor meter produced by Valley Polymerization Technology Co., Ltd. (Beijing, China) was used. The test voltage and current were set to 1.0 V and 10 μA, respectively, with the probe spacing set to 1.0 mm. The number of repeated measurements of single-point resistance was set to 10 times. The ToF-SIMS depth profiles were captured using ION-TOF GmbH (Münster, Germany) under a chamber pressure of below 5 × 10^−9^ mbar to analyze the content of Si, O, Cu, and C in each layer of the specimen before and after annealing. For high mass resolution analysis, the spectra mode with pulsed 30 keV Bi^3+^ (~0.48 pA pulsed current) ion beam was applied. The focused analysis area was 150 × 150 μm^2^, with 2 keV Cs ion beam sputtering at the same time, 400 × 400 μm^2^ sputter raster, and noninterlaced mode.

## 3. Results and Discussion

### 3.1. First Thermal Transformation and Corresponding Characterization

**Figure 2:**
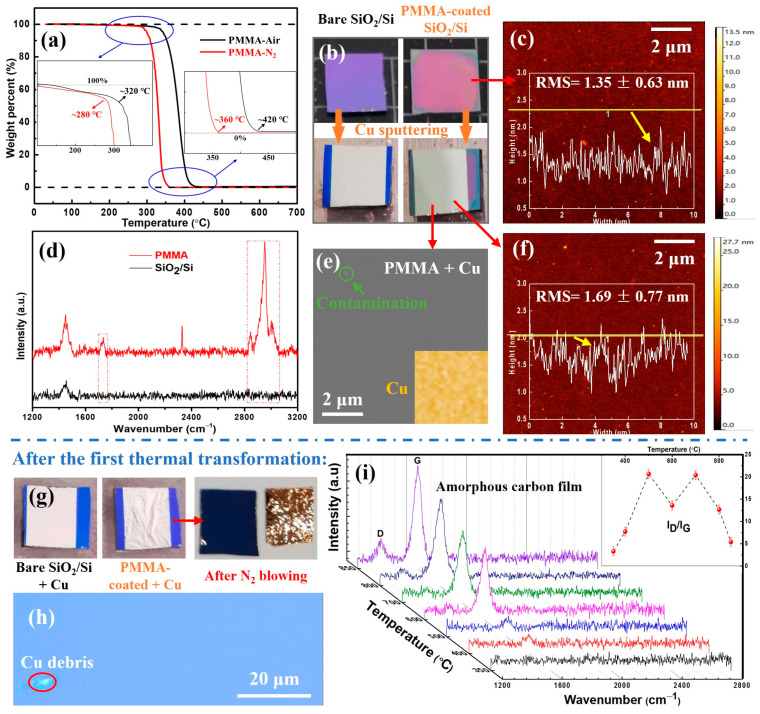
Characterization of raw materials and the amorphous film after the first thermal transformation process. (**a**) Thermogravimetric curves of PMMA particles in two different gaseous conditions. (**b**) Optical images of uncoated, PMMA-coated 300 nm SiO_2_/Si specimens and their copper coating state, respectively. (**c**) 2D AFM height image of the PMMA-coated specimen showing a flat surface with an average RMS of 1.35 ± 0.63 nm. (**d**) Raman spectrum of the uncoated and PMMA-coated substrates. (**e**) SEM photograph of the copper-coated specimen showing a homogeneous copper film. The inset EDS mapping of Cu element shows the specimen covered with uniform copper film and the contamination within the green circle is highlighted for focusing. (**f**) 2D AFM height image of the copper-coated specimen, revealing a slight surface roughness increase after copper sputtering. (**g**) Optical images of the uncoated and coated PMMA film substrates after thermal transformation. The copper film on the PMMA-coated substrate exhibits obvious corrugation and can be easily blown off with nitrogen gas. (**h**) Photograph of the thermal-transformed substrate after the copper film blown off; the copper debris within the red circle is highlighted for optical focusing. (**i**) Raman spectra of the obtained films on SiO_2_/Si substrates at different experiment temperatures after copper film removal; the inset is the corresponding D/G bands ratio.

The thermogravimetric analysis (TGA) of PMMA particles (Figure 2a) reveals that the thermal decomposition process is slightly affected by the atmospheric conditions; the onset and completion temperatures of the thermal decomposition occur earlier in high-purity nitrogen than in the air. However, the overall thermal decomposition process of PMMA remains consistent, and the decomposition is complete at approximately 420 °C (corresponding to ~360 °C in high-purity nitrogen and ~420 °C in air), with the final mass being reduced to zero. This indicates that the PMMA particles used in this experiment completely decompose at high temperatures (>420 °C) under free space and unprotected conditions. The spin-coated silicon oxide slices exhibit a different optical contrast than the initial substrate (Figure 2b). The copper-film-sputtered silicon oxide slices, with or without the PMMA film, also exhibit a consistent surface flatness, indirectly indicating that the PMMA film obtained through spin coating has high surface quality. The atomic force microscopy (AFM) height test result in Figure 2c shows that the surface of the PMMA-spin-coated silicon oxide slice is relatively flat, with a root mean square (RMS) roughness of 1.35 ± 0.63 nm, which directly proves that the PMMA film prepared in this experiment has high surface quality. After being scratched out of a trench by a tweezer, the thickness of PMMA thin film is 87.68 ± 1.25 nm under the AFM height test (Figure S1). The characteristic peaks in the Raman spectrum (Figure 2d) provide additional evidence of the successful high-quality spin coating of PMMA on silicon oxide slices. As shown in Figure 2e, the surface scanning electron microscopy (SEM) characterization of the copper-sputtered specimen shows that the copper film on the surface of the specimen is flat and clean, forming a good covering protection for the underlying PMMA film, and the energy-dispersive X-ray spectroscopy (EDS) pattern also confirms the uniform consistency of the copper film. The roughness of the PMMA-spin-coated specimens after copper sputtering is slightly increased compared with that before copper sputtering, and the RMS roughness is 1.69 ± 0.77 nm (Figure 2f). However, the overall surface roughness is consistent, indicating that the copper sputtering process does not negatively affect the quality of the PMMA film. The tiny peaks in the local area might have resulted from the sputtering collision and/or adsorption of airborne impurities. The above characterization results demonstrate that the samples prepared in this experiment have high surface quality, providing a solid foundation for subsequent thermal transformation experiments.

After the first thermal transformation, the morphology of the specimens with the spin-coated PMMA film changed significantly in comparison to the specimens without spin-coated PMMA (Figure 2g). The copper film on the surface of the sample without PMMA film did not undergo noticeable morphological changes after thermal transformation and remained flat and intact, demonstrating a tight bond with the underlying silicon oxide slice. However, the copper film on the surface of the sample with PMMA film underwent apparent morphological deformation. The surface copper film developed visible bulges and could be fully and cleanly separated from the substrate by nitrogen blowing, indicating that the copper film lost its good bond with the substrate after thermal transformation. The use of PMMA as a precursor for the preparation of graphene film on the surface of copper foil also supports this morphology change [37], which suggests that the PMMA film generates gaseous products during the thermal transformation process, leading to the loss of the PMMA film’s quality, and ultimately, the surface copper layer loses its tight bond with the substrate. The optical characterization of the substrate after blowing off the copper film revealed a uniform contrast, and no copper layer or other impurity residues were detected (Figure 2h). After a series of thermal transformation experiments, Raman spectra collected on the surface of the substrate respectively found a new product generated, which was analyzed as an amorphous carbon film, and the evolution of the amorphous carbon film had a certain correlation with the thermal transformation temperature. Figure 2i shows the Raman patterns on the surface of the silicon oxide slices after blowing off the surface copper film, in which two obvious characteristic peaks of carbon materials appear, located around 1350 cm^−1^ and 1580 cm^−1^, respectively. These characteristic peaks correspond to the defect D peak and graphite-type G peak of the amorphous carbon film. The evolution of the characteristic peaks of amorphous carbon film with thermal transformation temperature indicates that the amorphous carbon film only begins to grow at about 400 °C, and no corresponding characteristic peaks appear below this temperature. The temperature is almost the same as the complete thermal decomposition temperature of PMMA particles in Figure 2a, indicating that the amorphous carbon film originates from the decomposition of PMMA film. Furthermore, the Raman signal intensity of the amorphous carbon film gradually increases with the ascendance of temperature, which shows the accumulation of carbon on the surface. The D/G bands ratio indicates that the crystallization quality of the carbon film is improved when the temperature is higher than 700 °C. Although the characteristic peak intensity of the amorphous carbon film increased under the high-temperature thermal transformation, no new characteristic peak appeared, indicating that PMMA film under these thermal transformation conditions can only generate amorphous carbon film and cannot generate transfer-free graphene film as expected, which is different from some existing research results. This process can guide the preparation of transfer-free amorphous carbon films, but further research is needed to directly obtain graphene films on a dielectric substrate.

### 3.2. Second Thermal Transformation and Corresponding Characterization

**Figure 3:**
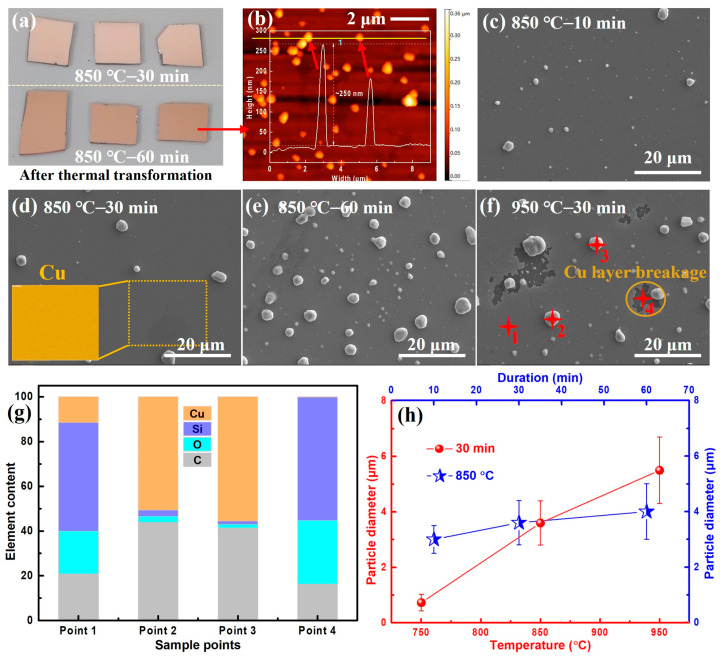
Surface morphology characterization of the copper film after the second thermal transformation process. (**a**) Macroscopic images of the transformed specimens under various thermal conditions show no apparent damage on the surface of copper film. (**b**) Surface 2D AFM height image of the thermal transformed specimen under 850 °C for 60 min reveals the presence of discrete particles on the copper film, with an average height of up to 250 nm. (**c**–**f**) Surface SEM photographs of the copper film under different transformation conditions, with the corresponding experiment condition marked in the upper-left corner. The inset in (**d**) and four red points in (**f**) display the EDS mapping spectrum of copper and EDS spot scanning positions, respectively. These images show varying amounts and distributions of particles on the transformed specimen’s surface. (**g**) Related element content captured from EDS spectra of the four points highlighted in (**f**). (**h**) Statistic analysis of the particle’s diameter on the sample’s surface under different transformation conditions.

According to the idea of the first thermal transformation of PMMA film, we carried out a second copper sputtering treatment on the obtained transfer-free amorphous carbon film and proceeded with a series of thermal transformation experiments again. The final morphology of these samples is shown in Figure 3a. It can be seen that the surface macro-morphology of the copper film is completely different from the PMMA-coated samples after the first thermal transformation but is similar to the sample without PMMA layer. The copper film after the second thermal transformation remains complete and flat, without obvious loss or bulges, showing a good protective effect of the substrate. Figure 3b is the 2D AFM image of the copper layer on the surface of the specimen after thermal transformation. The AFM height image shows that peak-like protrusions with different heights appear on the surface of the copper layer, indicating that the copper layer has undergone obvious morphological evolution under high-temperature conditions and formed copper particles with different sizes, which is also confirmed by subsequent SEM and EDS characterization.

Figure 3c–f shows the surface SEM photographs of the specimen with copper layer under different thermal transformation conditions, and it can be seen that granular products with different densities and size distributions appear on the surface of the copper layer. EDS results show that the main chemical composition of the particles is copper (Figure 3g), but due to the depth of the EDS detection, silicon and carbon elements in the underlying structure are also present. Unlike the surface granular products, the EDS spectrum in the plane area shows that silicon is the dominant chemical element, which mainly comes from the underlying silicon oxide substrate. It can be seen that the generation of granular products on the surface of the specimen is mainly attributed to the high-temperature “dewetting” of the surface copper layer, and there is an obvious dependence between the size of particles and thermal transformation conditions. Figure 3h shows the statistical curves of copper particles’ diameter under different thermal transformation conditions. It can be seen that under the same thermal transformation temperature, the size of copper particles increases with the time, and in the same way, under the same thermal transformation time, copper particles’ size and temperature are also positively correlated. Why does the copper layer appear the above “dewetting” phenomenon? According to previous research, specifically, the metal layer is thermally driven by energy minimization in a high-temperature environment, and the total energy must be minimized by increasing the surface energy of the system. The aforementioned “dewetting” transformation of the copper layer may be attributed to the same phenomenon of high-temperature “Ostwald ripening” in bulk metals.

The copper layer on the surface of the sample undergoes a “dewetting” phenomenon in the high-temperature environment, but it remains intact below the temperature of 850 °C and 60 min. The EDS mapping of the copper film in Figure 3d confirms the uniform distribution of copper on the surface. However, at 950 °C for 30 min, the surface copper layer shows not only “dewetted” particles with a larger average size but also some broken areas and exposure of the lower amorphous carbon film (Figure 3f). This indicates that the copper layer loses its protective effect for the lower amorphous carbon film under these severe thermal conditions. To ensure the surface quality of the amorphous carbon film and the second thermal transformation product, it is necessary to prevent the damage of the surface copper layer during the heating process. In our studies, the conditions for the second thermal transformation experiment were controlled within the range where no obvious damage occurs on the surface copper layer.

### 3.3. Structure and Properties of the Fully Processed Specimens

**Figure 4:**
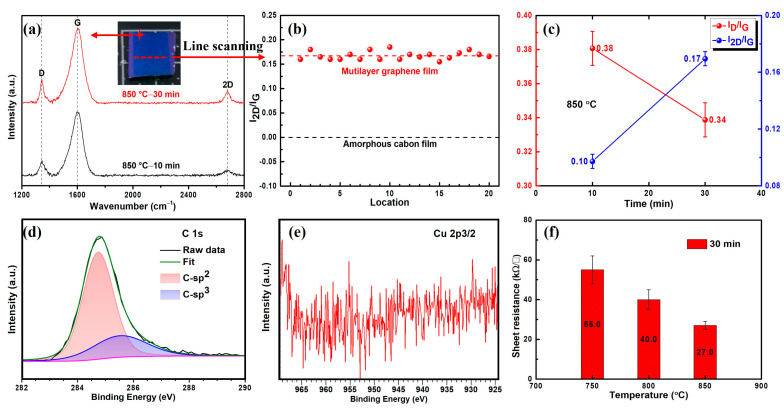
Properties of multilayer graphene characterized on SiO_2_/Si substrate after copper etching. (**a**) The typical Raman spectrum of transfer-free graphene film with different thermal transformation times is presented. The inset displays the macroscopic image of graphene film. (**b**) Raman intensity ratio (2D to G) on different locations is shown, demonstrating the homogeneous coverage of the graphene film and its high uniformity. (**c**) The statistical analysis of the Raman intensity ratio of 2D to G and D to G under different thermal transformation conditions is provided. (**d**–**e**) XPS C 1 s and Cu 2p_3/2_ spectra of the transfer-free graphene film are depicted. (**f**) The sheet resistance of the graphene film under different thermal transformation temperatures, measured by a four-probe system, is also presented.

The macroscopic topography photo of the silicon oxide slice after copper layer etching, shown in Figure 4a, clearly displays the distinct and flat carbon material film, demonstrating that the amorphous carbon film remained intact during the second thermal transformation process. This can be attributed to the intact coverage protection provided by the surface copper layer. The thickness of the obtained carbon film is 3.11 ± 0.86 nm under AFM height characterization (Figure S2) and is similar to the ~10-layer graphene film. Raman spectroscopy characterization revealed that, unlike the first thermal transformation product of PMMA film, which only displayed D and G peaks, the carbon material obtained after the second thermal transformation exhibits a typical graphene characteristic 2D peak (~2700 cm^−1^). The appearance of these characteristic peaks confirms the in situ transformation of amorphous carbon film into graphene, and the intensity ratio of 2D peak to G peak determines the multilayer structure of the transfer-free graphene film. The intensity of the 2D peak in the Raman spectrum also indicates that longer thermal transformation time results in graphene film with better crystallization quality. Evaluating the uniformity of the intensity ratio of Raman 2D peaks to G peaks within a certain area of graphene film is another effective way to evaluate the surface uniformity of graphene. Figure 4b displays the I_2D_/I_G_ result of the red dashed line in Figure 4a, which reveals that the average I_2D_/I_G_ ratio of the graphene film obtained in this process is around 0.16, demonstrating that the graphene film has good uniformity. Figure 4c shows that, under different thermal transformation times, the longer transformation time results in higher quality multilayer graphene film. The acquisition of this transfer-free multilayer graphene film can contribute to the convenience of direct integration of graphene film in the field of graphene-based semiconductor devices.

In addition to the surface Raman spectroscopy, X-ray photoelectron spectroscopy (XPS) can also provide us with more structural details of the transfer-free graphene films. Meanwhile, by analyzing the bonding type of chemical elements, we can also determine the impurity binding of the graphene film and evaluate the overall quality of the graphene film. XPS results of in situ growth of multilayer graphene films on silicon oxide substrate are shown in Figure 4d,e, where Figure 4d shows the XPS spectra of the C 1 s range, from which a significant peak is evident at 284.80 eV, as well as a broadened peak around 285.40 eV. After theoretical analysis, the peak position of 284.80 eV in C 1 s spectrum corresponds to the double bond structure of the carbon element, indicating that the carbon element in the transfer-free multilayer graphene film is mainly asp^2^-type structure, which is also the typical structure in graphene film. The peak position at 285.40 eV mainly corresponds to the carbon sp^3^ bonding structure, which shows that there are some defects in the multilayer graphene film and is also one reason for the apparently defective D peak in the Raman spectrum in Figure 4a. The lack of apparent peaks in the Cu 2p_3/2_ region (Figure 4e) testifies that the etching solution used in our experiment has efficient copper etching capability and can obtain a clean and lossless graphene film.

Since the multilayer graphene film obtained in this project is in situ grown on the silicon oxide substrates, the traditional physical transfer process is avoided, and the electrical four-probe test is particularly convenient. The electrical results show that the surface resistance of the transfer-free multilayer graphene film is below 30 kΩ/□, which is similar to the nitrogen-doped graphene film prepared in the reference [38]. Though the electrical properties of the obtained transfer-free graphene film are relatively inferior, there are still some application scenarios that do not require extremely low resistance, such as LED or THz device manufacturing [22,39]. In addition, from Figure 4f, we can see that the final obtained transfer-free multilayer graphene film shows a significantly improved trend of sheet resistance with the thermal transformation temperature increase. This phenomenon also demonstrates that the transformation process of graphene film without transfer is a thermodynamic driving process. Under the thermodynamic drive caused by high temperature and the catalysis of surface metal copper, carbon atoms can be effectively assembled into a graphene structure. The higher temperature translates to the higher crystallization quality of the graphene film, which in turn makes its electrical properties significantly enhanced. On the other hand, a possible factor resulting in the weak electrical properties of multilayer graphene obtained in this project comes from the multiple defects that lead to severe scattering of charge carriers in graphene film during electrical transport, and this can be verified by defective D peaks in Raman spectrum and carbon sp^3^ binding bonds in XPS C 1 s spectrum.

### 3.4. Mechanism of the Transfer-Free Graphene Synthesis

**Figure 5:**
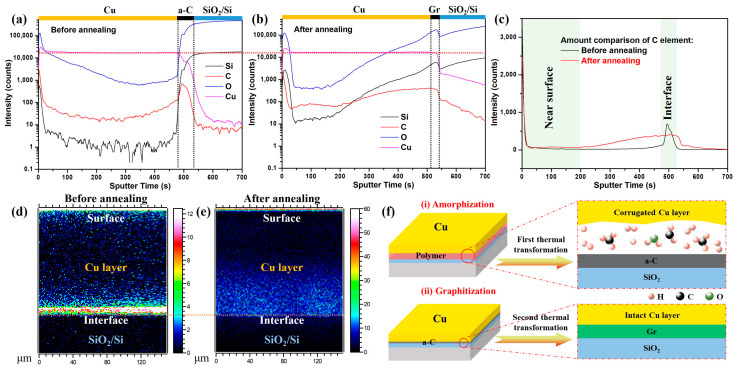
ToF-SIMS results of specimen before and after the second thermal transformation and corresponding mechanism. (**a**,**b**) The ToF-SIMS depth profiles of the specimen before and after thermal annealing with copper layer. (**c**) The comparison of the carbon element’s depth profile of the specimen before and after annealing. Cross-sectional images of the 2D depth profile of the carbon element of the specimen before (**d**) and after (**e**) annealing. (**f**) A schematic diagram of the mechanism of transfer-free graphene growth by two steps: (i) amorphization process of the spin-coated PMMA film induced by thermal decomposition and copper film covering, and (ii) graphitization process of the amorphous carbon film induced by copper catalytic film resulting in in situ synthesis of transfer-free graphene film on SiO_2_/Si substrate.

By comparing the first and second thermal transformation results, we can clearly see that the copper layer plays significantly different roles in the two thermal transformation processes, and both of these roles are indispensable parts of the synthesis of transfer-free multilayer graphene film. Reference [37] investigated the mechanism of solid PMMA particles participating in the growth of high-quality graphene films on the surface of copper foil. They pointed out that solid PMMA will first form gas-phase groups dominated by CH_3_^+^, H_2_, and H_2_O through the thermal decomposition process, and then gaseous carbon radicals will deposit and stitch on the surface of copper foil to grow graphene films. It is apparent that the polymer carbon source PMMA will undergo gas phase decomposition under high-temperature pyrolysis conditions, resulting in the loss of mass. Therefore, during the first thermal decomposition process in our project, the copper layer on the surface of the specimen loses its original support due to the loss of gaseous products of the lower PMMA layer, and then obvious wrinkles and bulges occur, and the copper layer is easily blown away by gas. It should be mentioned that, based on the structural characterization of the obtained products on the silicon oxide substrate after blowing away the copper layer, it is found that due to the physical barrier of the surface copper layer, the PMMA layer does not undergo complete and thermal pyrolysis with no solid residues, like in the free space, but generates amorphous carbon film on the substrate through amorphous transformation (see Figure 5(fi)).

Unlike the PMMA pyrolysis process, the amorphous carbon film does not experience gaseous phase transformation during the second thermal transformation, so the surface copper layer remains intact after the thermal annealing. In order to deeply study the specific changes of each element in this process, the content distributions of Si, O, Cu, and C elements before and after thermal transformation were analyzed by ToF-SIMS, and the results are shown in Figure 5a–e. From Figure 5a,b, we can see that the four elements underwent different degrees of mutual diffusion at the interface after annealing. The content of the surface copper layer was negligible and unchanged after annealing and showed high temperature stability. The diffusion behavior of carbon can be traced in Figure 5c–e; after thermal annealing, the interfacial carbon element diffused to a certain extent into the bulk copper layer, but it can be clearly seen that it failed to penetrate to reach the surface of the copper layer and mainly aggregated at the interface, which is an important prerequisite for the formation of the interfacial graphene film.

Simplified thermodynamic calculations show that the energy difference before and after the second thermal transformation can be expressed as $∆G=\left[\gamma_{Cu}+\gamma_{Cu/Gr}+\gamma_{Gr/{SiO}_{2}}+t∆G_{trans}\right]-[\gamma_{Cu}+\gamma_{Cu/a-C}+\gamma_{a-C/{SiO}_{2}}]$; here, *γ* is the interfacial energy, *t* is the graphene thickness at the interface, and Δ*G_trans_* is the graphene formation energy per unit volume with copper catalyzed, respectively [15,40]. The energy change of interfacial graphene under this condition is −20.16 J/m^2^, which indicates that it is more thermodynamically favorable to synthesize graphene film at the interface. Therefore, under the protection and catalyzation of the copper layer, graphitization transformation occurs, and the graphene multilayer film is ultimately generated (Figure 5(fii)).

## 4. Conclusions

In summary, this study investigated the two-step thermal transformation process of PMMA polymer to obtain transfer-free graphene film on dielectric substrates, utilizing film spin coating, copper layer sputtering, and wet etching techniques. The microstructural evolution of the PMMA film layer was examined, providing valuable insights for in situ graphene film synthesis. The copper layer plays a crucial role in the physical protection and catalytic crystallization processes, and precise control of its structural parameters is important for obtaining high-quality transfer-free graphene film. The two-step thermal transformation process successively yields clean, flat transfer-free amorphous carbon film as low as 400 °C and multilayer graphene film at 850 °C, which is more economical and energy-saving than the nickel-catalyzed method. The quality of these films can be optimized by controlling the initial spin coating and the thermal annealing parameters. Our study deepens the understanding of copper-assisted polymer transformation for graphene growth, without the need for post-transfer procedures. This approach has potential in compatible semiconductor processes and can offer more choices for graphene-based functional device manufacturing.

## Data Availability

The data in this study are available upon request.

## Supplementary Materials

The following supporting information can be downloaded at: https://www.mdpi.com/article/10.3390/ma16165603/s1, Figure S1: Thickness characterization of the PMMA thin film; Figure S2: Characterization of the obtained multilayer graphene film.
