# Supplementary material for: Two-Step Thermal Transformation of Multilayer Graphene Using Polymeric Carbon Source Assisted by Physical Vapor Deposited Copper
Source: Materials (Basel). 2023 Aug 13;16(16):5603. doi: 10.3390/ma16165603 (PMC10456611; doi:10.3390/ma16165603)
Supplement: Supplementary file 1 [file materials-16-05603-s001.zip › materials-2531746-supplementary.pdf]

Supplementary Information for

Two-step thermal transformation of multilayer graphene using polymeric carbon source assisted by physical vapor deposited copper

Yong Huang, Jiamiao Ni, Xiaoyu Shi, Yu Wang, Songsong Yao, Yue Liu* and Tongxiang Fan*

State Key Laboratory of Metal Matrix Composites, School of Materials Science and Engineering, Shanghai Jiao Tong University, Shanghai 200240, China

***Correspondence:** Yue Liu, yliu23@sjtu.edu.cn (Y.L.); Tongxiang Fan, txfan@sjtu.edu.cn (T.F.)

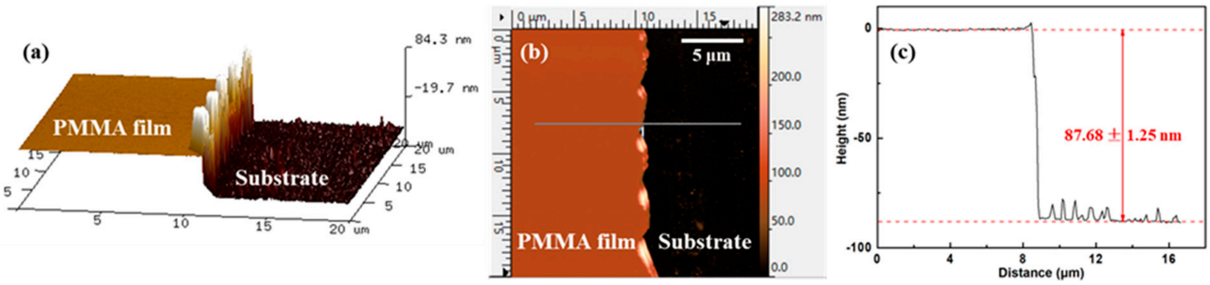

Figure S1. Thickness characterization of the PMMA thin film. **(a)** 3D and 2D AFM **(b)** image of the PMMA film scratched by a tweezer. **(c)** AFM height curve of the PMMA film corresponding to the "line 1" in **(b)**.

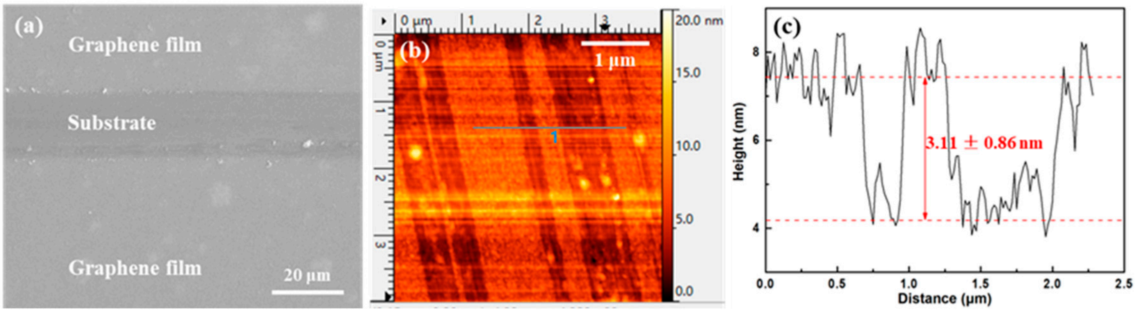

Figure S2. Characterization of the obtained multilayer graphene film. **(a)** SEM and **(b)** AFM height image of the graphene film scratched by a tweezer. **(c)** AFM height curve of the graphene film corresponding to the "line 1" in **(b)**.
